# High-throughput malaria serosurveillance using a one-step multiplex bead assay

**DOI:** 10.1186/s12936-019-3027-0

**Published:** 2019-12-04

**Authors:** Eric Rogier, Lotus van den Hoogen, Camelia Herman, Kevin Gurrala, Vena Joseph, Gillian Stresman, Jacquelin Presume, Ithamare Romilus, Gina Mondelus, Tamara Elisme, Ruth Ashton, Michelle Chang, Jean F. Lemoine, Thomas Druetz, Thomas P. Eisele, Alexandre Existe, Jacques Boncy, Chris Drakeley, Venkatachalam Udhayakumar

**Affiliations:** 1 Malaria Branch, Division of Parasitic Diseases and Malaria, Centers for Disease Control and Prevention, Atlanta, GA 30329 USA; 2 Department of Infection Biology, London School of Hygiene & Tropical Medicine, London, WC1E 7HT UK; 3 CDC Foundation, Atlanta, GA 30308 USA; 4 Georgia Institute of Technology, Atlanta, GA 30332 USA; 5 Center for Applied Malaria Research and Evaluation, Department of Tropical Medicine, Tulane University School of Public Health and Tropical Medicine, New Orleans, LA 70112 USA; 6 Laboratoire National de Santé Publique (LNSP), Ministère de la Santé Publique et de la Population (MSPP), Port-au-Prince, Haiti; 7 Programme National de Contrôle de la Malaria/MSPP, Port-au-Prince, Haiti; 8 Department of Social and Preventive Medicine, University of Montreal School of Public Health, Montreal, QC H3X 1X9 Canada

**Keywords:** Malaria, Multiplex immunoassay, Seroprevalence, Protocol

## Abstract

**Background:**

Serological data indicating the presence and level of antibodies against infectious disease antigens provides indicators of exposure and transmission patterns in a population. Laboratory testing for large-scale serosurveys is often hindered by time-consuming immunoassays that employ multiple tandem steps. Some nations have recently begun using malaria serosurveillance data to make inferences about the malaria exposure in their populations, and serosurveys have grown increasingly larger as more accurate estimates are desired. Presented here is a novel approach of antibody detection using bead-based immunoassay that involves incubating all assay reagents concurrently overnight.

**Results:**

A serosurvey in was performed in Haiti in early 2017 with both sera (n = 712) and dried blood spots (DBS, n = 796) collected for the same participants. The Luminex^®^ multiplex bead-based assay (MBA) was used to detect total IgG against 8 malaria antigens: PfMSP1, PvMSP1, PmMSP1, PfCSP, PfAMA1, PfLSA1, PfGLURP-R0, PfHRP2. All sera and DBS samples were assayed by MBA using a standard immunoassay protocol with multiple steps, as well a protocol where sample and all reagents were incubated together overnight—termed here the OneStep assay. When compared to a standard multi-step assay, this OneStep assay amplified the assay signal for IgG detection for all 8 malaria antigens. The greatest increases in assay signal were seen at the low- and mid-range IgG titers and were indicative of an enhancement in the analyte detection, not simply an increase in the background signal of the assay. Seroprevalence estimates were generally similar for this sample Haitian population for all antigens regardless of serum or DBS sample type or assay protocol used.

**Conclusions:**

When using the MBA for IgG detection, overnight incubation for the test sample and all assay reagents greatly minimized hands-on time for laboratory staff. Enhanced IgG signal was observed with the OneStep assay for all 8 malaria antigens employed in this study, and seroprevalence estimates for this sample population were similar regardless of assay protocol used. This overnight incubation protocol has the potential to be deployed for large-scale malaria serosurveys for the high-throughput and timely collection of antibody data, particularly for malaria seroprevalence estimates.

## Background

Population-level detection of host antibodies has allowed estimates for transmission intensity [1–3], infection prevalence [4, 5], disease burden [6], and population immunity for vaccine preventable diseases [7]. For greatest generalizability and epidemiologic utility, serosurveys benefit from use of population-based probabilistic sampling methods and are strengthened when increased proportions of the population are sampled—inherently generating more statistical power and accurate estimates [8]. Serological data are increasingly desired as supporting evidence for verifying the interruption of transmission and infectious disease elimination from a population [7, 9], and serosurveys that include laboratory testing for multiple diseases allow efficient monitoring of impact across programmes. Specifically for *Plasmodium* spp. parasites, the presence of IgG antibodies against malaria antigens has been used as an indicator to generate point estimates for malaria transmission as well as to monitor changes in malaria burden in a population over time [10–12].

Antibody detection assays have evolved substantially over from detecting whether serum antibodies are present at a titer defined as “positive”, to providing quantitative estimates of antigen-specific antibodies present in a sample. Recently, multiplex bead-based immunoassays (MBAs) have further expanded antibody detection efforts, having the ability to assay for multiple targets simultaneously in the same well. This strategy has increased the feasibility of implementing integrated disease serosurveys [13, 14], with the additional benefit that the MBA has been shown to be more sensitive than ELISA for analyte detection of some targets [15–17]. As with the ELISA, current MBA protocols call for sequential steps of incubation with sample, incubation with a secondary (or detection) antibody, and incubation with a reporter that will provide a quantitative assay signal. Alternative protocols have also used secondary antibodies directly linked to a reporter [16]. In performing these steps in this particular order, the researcher is able to ensure the signal above background generated by the immunoassay is a true signal reflective of the presence of the analyte. Here is presented a modification to the MBA that adapts the assay protocol to involve incubation of the sample and all reagents concurrently. This “OneStep” assay is formatted to include overnight incubation at room temperature, allowing for minimal hands-on time by laboratory staff, and obviating the need for washes between sequential incubation steps. To directly compare IgG both the antibody detection capacity of the standard and OneStep MBA protocols and estimates that would be generated for an actual serosurvey, 8 antigens from three *Plasmodium* species (*Plasmodium falciparum*, *Plasmodium vivax*, and *Plasmodium malariae*) were tested on a sample set of sera (n = 712) and dried blood spots (DBS, n = 796) collected from the same participants in a 2017 serosurvey in Haiti.

## Methods

### Human subjects and blood samples

Laboratory staff did not have access to personal identifiers. Study participants consented for a diagnostic test and blood sample collection that would assay for markers of malaria. The field survey received approval from the Haitian Ministry of Public Health and Population Bioethics Committee (Comité National de Bioéthique) (#1516-30), and the Institutional Review Boards (IRBs) of Tulane University (#794709), and the London School of Hygiene & Tropical Medicine (#10393). Current laboratory activity was not considered to constitute engagement in human subjects research by the CDC Center for Global Health Office of the Associate Director for Science (#2016-135a). Consent for children (< 18 years) was provided by a parent or guardian and children above 6 years gave assent to participate. Individuals aged 16 or 17, who were married, head of household or a parent were considered ‘mature minors’ and consented directly. Thumbprint consent or assent (countersigned by a witness) was used for illiterate participants. Individuals under 6 months of age or requiring immediate medical attention were excluded. A single finger-prick was performed on consenting participants to collect capillary blood (Safe-T-Fill™ Capillary Blood Collection Systems: EDTA, RAM Scientific Inc.) for spotting of whole blood on filter paper (Whatman 903, GE Healthcare); remaining blood stored at 4 °C for later serum fractionation. Study participants also had a malaria rapid diagnostic test (RDT, SD Bioline Malaria Antigen P.f.; 05FK50), and individuals with a positive RDT result received free treatment as per the national policy in Haiti. Serum and dried blood spot (DBS) samples (n = 796 DBS with 712 paired serum) were collected in April and May 2017.

At the Haitian national laboratory (*Laboratoire National de Santé Publique*, LNSP), blood remaining in the tube was centrifuged (5000*g* for 2 min) to fractionate and allow removal of serum. Blood dried on filter paper (dried blood spots, DBS) were eluted in Buffer B (PBS containing 0.5% BSA, 0.05% Tween 20, 0.02% sodium azide, 0.5% polyvinyl alcohol, 0.8% polyvinylpyrrolidone and 0.5% w/v *Escherichia coli* extract) by incubation overnight at 4 degrees. Whole blood elution from filter paper was done as to achieve a 1:50 whole blood dilution, which approximates a serum dilution of 1:100 with the assumption of 50% haematocrit in whole blood. Liquid serum was directly diluted 1:100 in Buffer B.

### Antigens and couplings

A summary of the 8 malaria antigens and the control antigen is displayed in Table 1. Recombinant *Schistosoma japonicum* glutathione-*S*-transferase (GST) protein coupled to a bead was used as a generic protein non-binding control. The 19 kD fragment of the *Plasmodium* merozoite surface protein 1 (MSP1) antigens for *P. falciparum*, *P. vivax*, and *P. malariae* have all been described previously [18]. The *P. falciparum* apical membrane antigen 1 (AMA1), circumsporozoite protein (CSP), and liver stage antigen 1 (LSA1) antigens have also been described in previous studies from our group [19, 20]. The 19-amino acid glutamate-rich protein R0 (GLURP-R0) fragment was synthesized as described previously [21], and the histidine-rich protein 2 (HRP2) Type A and Type B recombinant antigens were produced by Microcoat (Bernried am Starnberger See, Germany). For coupling of the HRP2 antigen to the microspheres, a 1:1 mixture was made of the Type A and Type B antigens so that equivalent amounts of these would be bound to the beads. All antigens were covalently linked to MagPlex (magnetic) microspheres (Luminex Corp., Austin, TX) as described previously [20]. Briefly, beads were pulse vortexed, transferred to a microcentrifuge tube and centrifuged for 1.5 min at 13,000*g*. Supernatant was removed and beads were washed with 0.1 M sodium phosphate, pH 6.2 (NaP). Beads were activated by suspending in NaP with 50 mg/mL of 1-ethyl-3-[3-dimethylaminopropyl]carbodiimide hydrochloride (EDC) and 50 mg/mL sulfo-NHS (sulfo *N*-hydroxysulfosuccinimide) and incubating with rotation for 20 min at room temperature (RT) protected from light. After wash with antigen-coupling buffer (4-morpholineethanesulfonic acid, MES; Sigma, St. Louis, MO), beads were suspended in antigen coupling buffer with the appropriate concentration of antigen (Table 1) and rotated for 2 h at RT protected from light. Beads were washed with PBS and suspended in PBS with 1% bovine serum albumin (BSA; Sigma) and incubated for 30 min at RT by rotation. Beads were then washed with storage buffer (PBS, 1% BSA, 0.02% sodium azide and 0.05% Tween-20) and suspended in storage buffer containing protease inhibitors (200 µg/mL Pefabloc, 200 µg/ml EDTA, 1 µg/mL pepstatin A and 1 µg/mL leupeptin) and stored at 4 °C.

**Table 1:** Antigens used in the study multiplex panel

Antigen alias	Organism	Tag	Source	Antigen coupling concentration (per 1.25 × 10^7^ beads)
PfMSP1-19	*Plasmodium falciparum*	GST	Recombinant, CDC	30 µg in MES pH 5
PvMSP1-19	*Plasmodium vivax*	GST	Recombinant, CDC	30 µg in MES pH 5
PmMSP1-19	*Plasmodium malariae*	GST	Recombinant, CDC	30 µg in MES pH 5
PfCSP	*Plasmodium falciparum*	GST	Peptide, CDC	30 µg in MES pH 5
PfAMA1	*Plasmodium falciparum*	GST	Recombinant, CDC	20 µg in MES pH 5
PfLSA1	*Plasmodium falciparum*	–	Peptide, CDC	60 µg in MES pH 5
PfGLURP-R0	*Plasmodium falciparum*	–	Peptide, CDC	30 µg in MES pH 5
PfHRP2	*Plasmodium falciparum*	GST	Recombinant, MicroCoat	25 µg in MES pH 5
glutathione-*S*-transferase	*Schistosoma japonicum*	–	Recombinant, CDC	15 µg in MES pH 5

### Bead-based immunoassay protocols

**Fig. 1:**
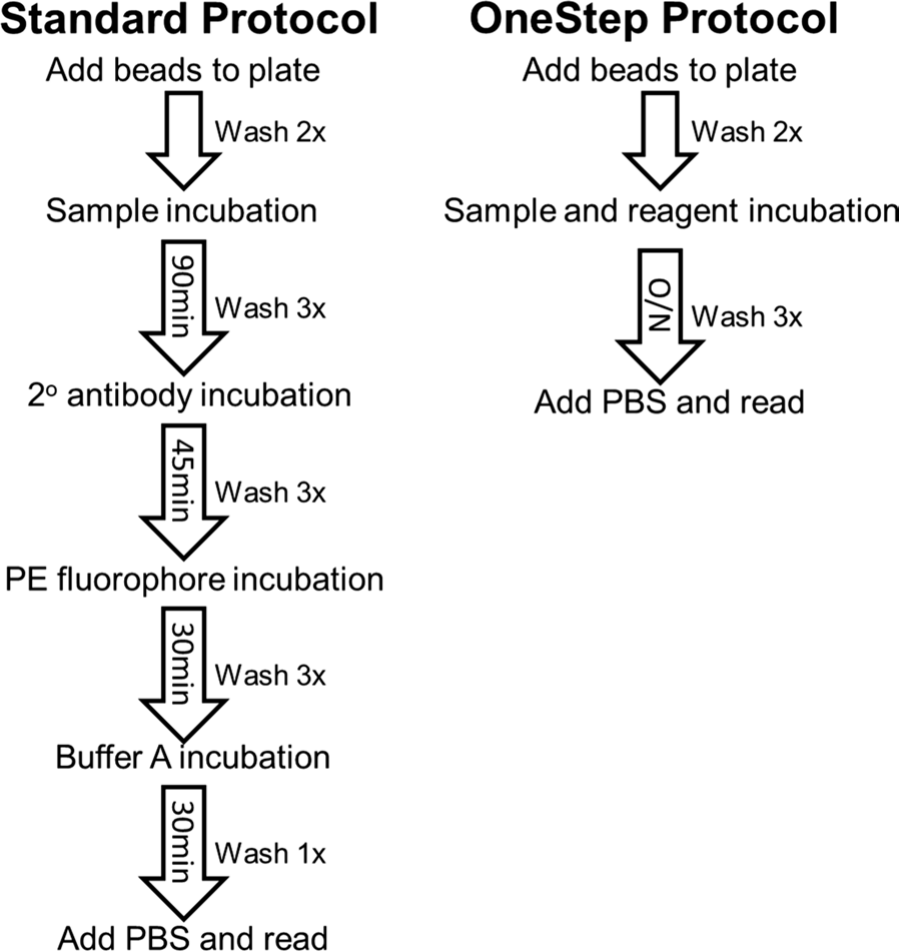
Protocols for standard and OneStep MBA. Each step of assay protocols is outlined with incubation time between steps indicated in the arrows

The standard MBA protocol was performed as described previously [20], and illustrated in Fig. 1. Briefly, the standard assay was performed in flat bottom BioPlex Pro 96 well plates (Bio-Rad, Hercules, CA) with washes between incubation steps used a handheld magnet (Luminex Corp). For wash steps, after addition of 100 µL wash buffer (PBS + 0.05% Tween-20, PBST) to each well, wash buffer was left in each well for one minute and magnet gently tapped to allow bead magnetization before inverting the plate to evacuate the wells of liquid. Beads (62,500 beads/antigen/plate) were suspended in Buffer A (PBS, 0.5% BSA, 0.05% Tween-20, 0.02% NaN_3_) and 50 µL bead mastermix added to each well. Plates were washed two times with PBST and 50 µL of diluted sample (as described above) was added to each well and incubated with shaking at room temperature for 90 min. After 3 washes with PBST, beads were incubated with 50 µL biotinylated detection antibody mix consisting of: anti-human IgG (1:500, 9042-08, Southern Biotech, Birmingham, AL) and anti-human IgG_4_ (1:625, 9200-08, Southern Biotech). Plates were incubated for 45 min and washed three times with PBST. 50 µL of streptavidin conjugated to phycoerythrin (PE) (1:200, S866, Invitrogen, Waltham, MA) was added to detect bound secondary antibody. After a 30-min incubation, wells were washed three times with PBST and incubated in Buffer A for 30 min under light shaking to remove any loosely bound antibodies. Samples were resuspended in 100 µL PBS and shaken for 30 min to resuspend beads; fluorescence data were collected immediately on the MAGPIX with Bio-Plex Manager™ MP software (BioRad) with a target of 50 beads per region per well. Median fluorescence intensity (MFI) signal was generated for a minimum of 50 beads/region, and background MFI from wells incubated with Buffer B was subtracted from each sample to give a final value of MFI minus background (MFI-bg) for analysis.

The OneStep assay was performed (as illustrated in Fig. 1) with the same samples and reagents and concentrations used in the standard MBA protocol. In 5 mL Buffer A, a bead mastermix was prepared with all regions included, and 50 µL bead mix was pipetted into a BioPlex Pro plate. Beads were washed 2× with 100 µL PBST, and 50 µL reagent mix (in 5 mL Buffer A same dilution of reagents: 1:500 anti-human IgG, 1:625 anti-human IgG_4_, 1:200 streptavidin-PE) was added to all wells, then 50 µL samples (or controls) were added to the reagent mix in the appropriate wells. Plates were incubated overnight with gentle shaking at room temperature and protected from light. The next morning (after ~ 16 h total incubation time), plates were washed 3×, and beads resuspended with 100 µL PBS and read on the MAGPIX machine. MFI signal was generated for a target of 50 beads/region, and background MFI from wells incubated with Buffer B was subtracted from each sample to give a final value of MFI-bg. Comparison in incubation times was accomplished by using a dilution curve of hyperimmune sera pooled from different areas of the world endemic for *P. falciparum* malaria.

### Statistical analysis

Statistical analyses were performed in SASv9.4 (SAS Institute, Cary, NC). Direct comparisons between MFI-bg values using the two protocols were represented by *k*-nearest-neighbor-based local regression (LOESS) curves created through the SGPLOT procedure with cubic interpolation and a degree of 2, and 95% confidence limits. Log-transformed MFI-bg values were fit to a two-component finite mixture model by the FMM procedure with normal distribution and maximum likelihood estimation outputs for component means and variances.

## Results

### Serum and DBS samples from serosurvey assayed by both protocols

**Fig. 2:**
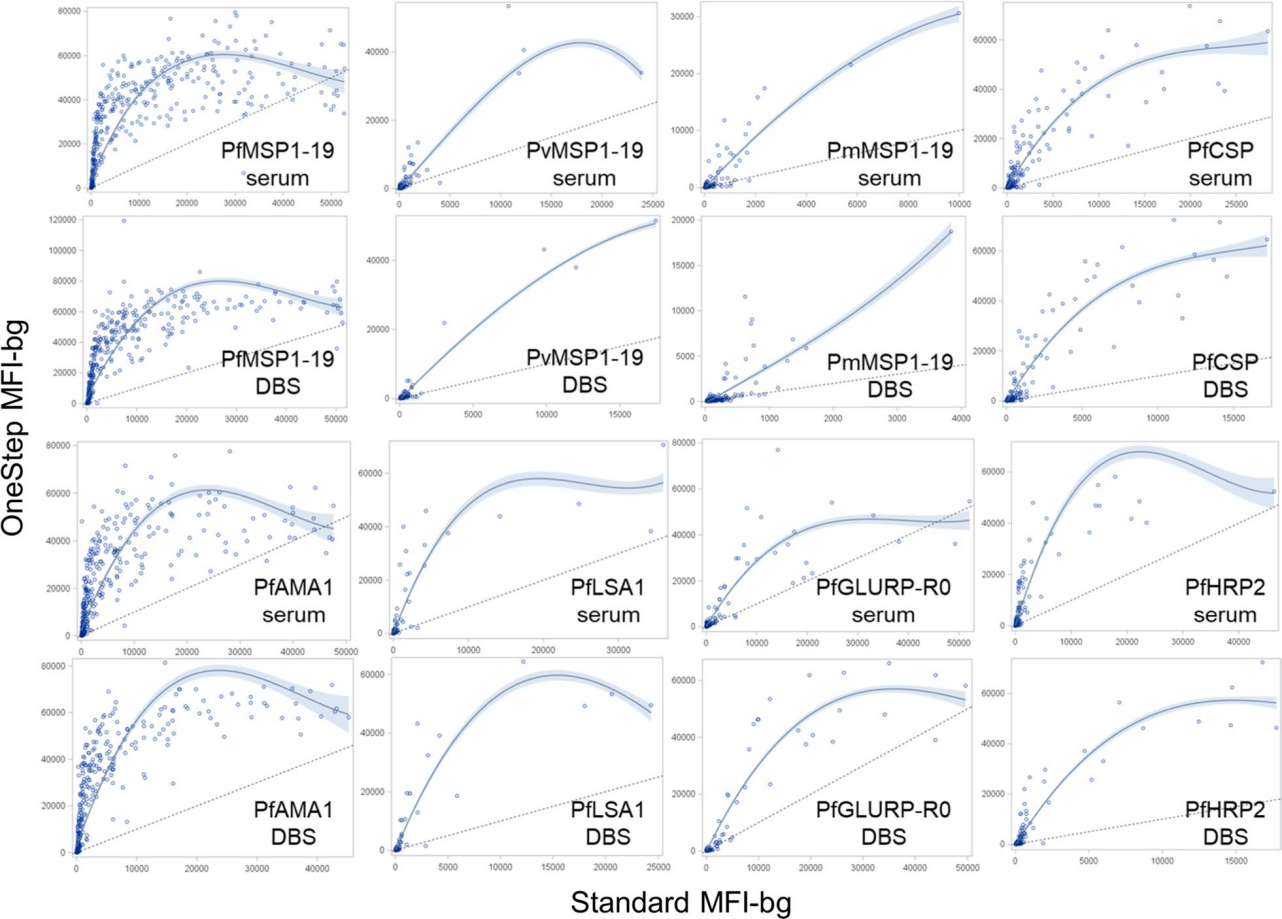
Comparison of assay median fluorescence intensity minus background (MFI-bg) signal by both assay protocols and sample types. Relationship in MFI-bg values for the eight malaria antigens between two protocols and two sample types visualized as LOESS curves with cubic interpolation and 95% confidence limits in shading, with y = x reference as a hashed line

Scatterplots comparing the MFI-bg values for the same person’s serum and DBS sampled assayed with both protocols are shown for all antigens in Fig. 2 with non-parametric LOESS curves and 95% confidence limits fit to the assay signal. In comparison to the y = x reference line which would indicate no change in assay signal between protocols, consistent increases in MFI-bg signal were observed for all antigens when using the OneStep protocol. As shown by the shape of each LOESS curve, the signal increase did not occur in a linear fashion over the range of IgG levels, and many of these curves mirror the shape of an exponential cumulative distribution function (CDF) with a rapid increase that eventually plateaus. These signal increases were most pronounced at the lower and mid-range IgG signals, and the highest IgG assay signals equivalent for both assay protocols for the PfMSP1-19, PfAMA1, and PfGLURP-R0 antigens. For the other 5 antigens, even at the highest IgG levels, the MFI signal for the OneStep protocol was augmented when compared to the highest MFI signals for same sample as assayed with the standard protocol.

**Fig. 3:**
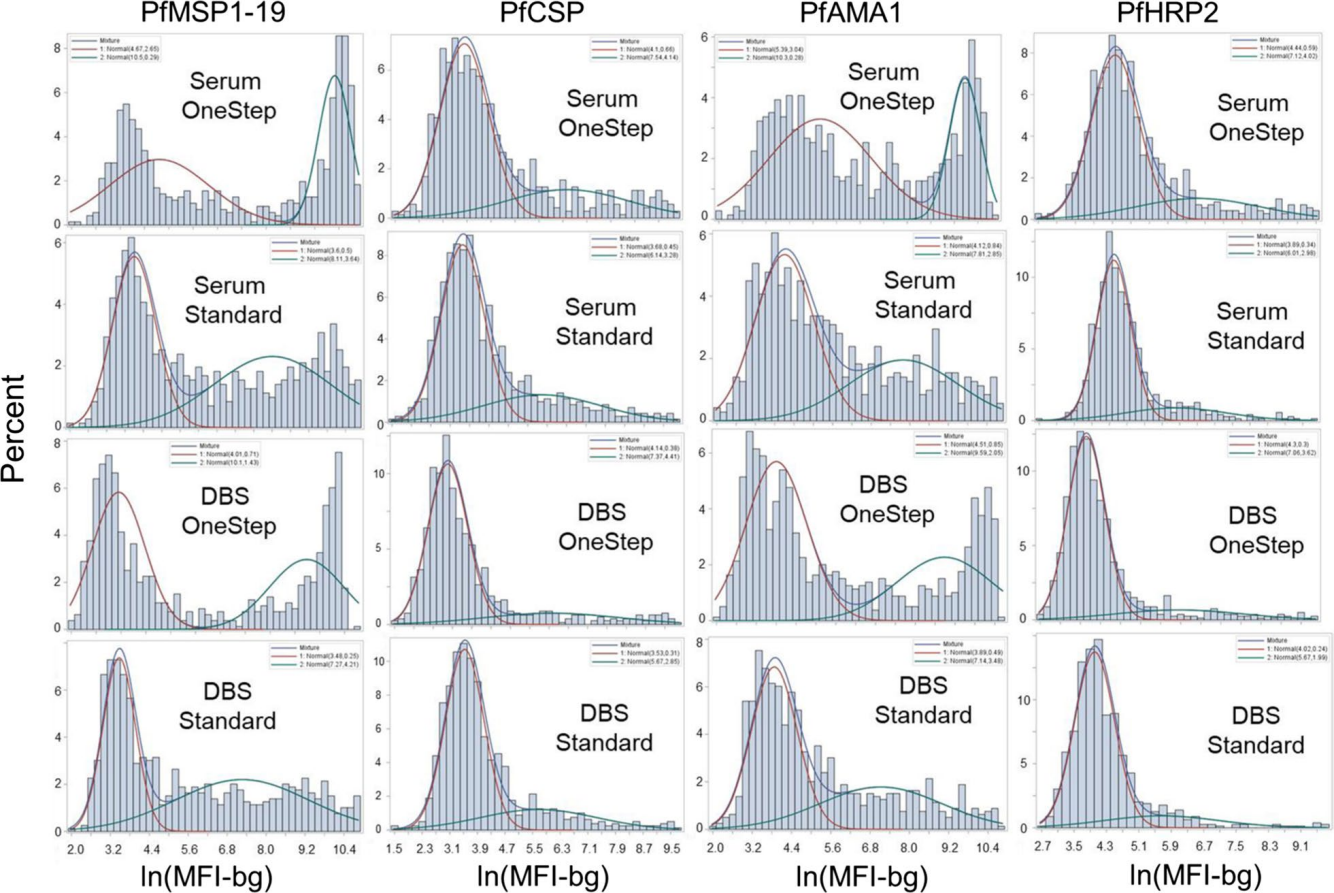
Fittings to two-component finite mixture models for antigen data collected by both assay protocols and sample types. Histograms are displayed for log-transformed MFI-bg values for the four most immunogenic malaria antigens as fit to a two-component finite mixture model. On each panel, estimates for lognormal mean and variance are displayed for the two components, and this information is included in Additional file 2. Plots for the remaining four antigens shown in Additional file 1

**Fig. 4:**
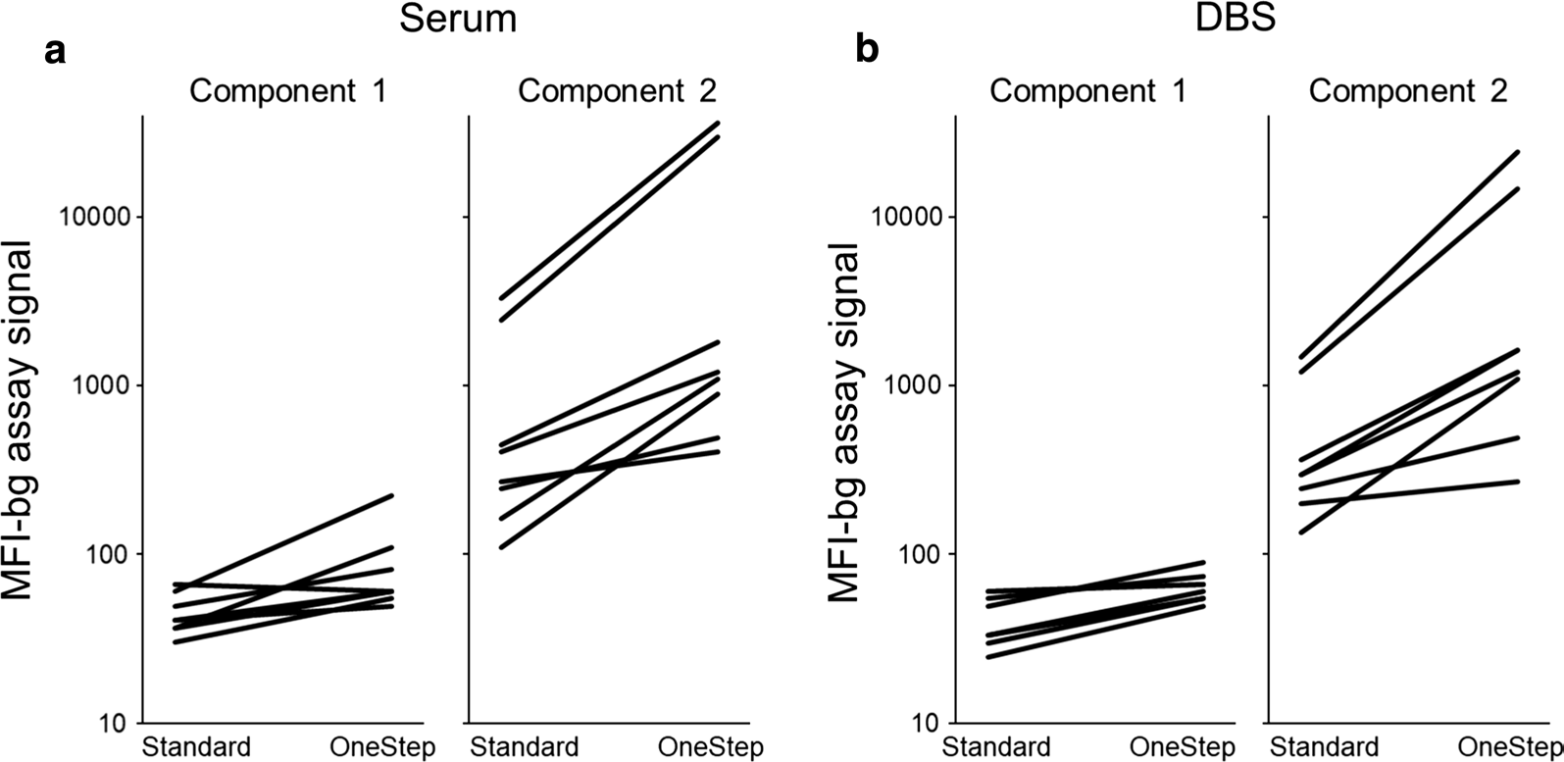
Change in MFI-bg means for first and second components of finite mixture model. Estimated means for each component were compared between the two assay protocols and the two sample types for all eight malaria antigens. Outputs for modeled lognormal means and variances displayed in Additional file 2

For the Haiti data, the 2-component finite mixture model (FMM) was used to estimate the antibody distributions in two putative subpopulations for each antigen: seronegative (the first, left-most component), and seropositive (the second, right-most component) [22, 23]. This statistical approach was chosen as it could be applied to the MFI-bg data for all antigens used in this study, and the maximum likelihood estimation (MLE) parametric outputs could be directly compared between the two protocols and sample types. Visualizations of the 2-component FMM plots for each antigen’s MFI-bg signals between the two protocols are shown in Fig. 3 for the four most immunogenic antigens and in Additional file 1 for the remaining antigens. The MLE outputs for lognormal means and variance of these components are shown in Additional file 2. Figure 4 shows the change between protocols in the estimated means of the modeled components for both sample types. Sample type did not appear to have a dramatic difference in the estimated means for the first or second components, but the OneStep assay protocol increased these means for both sera and DBS data. Minor increases were seen with OneStep protocol for the first component; the second component mean was substantially increased for most antigens (Fig. 4, Additional file 2). For both the sera and DBS data, a > 10-fold increase in the MFI-bg signal for the modelled mean of the second component was seen for PfMSP1-19 and PfAMA1, and a 1.5- to 8.2-fold increase observed for the other six antigens. When observing the assay signal to the generic GST antigen (which served as a non-binding internal control for this multiplex panel), neither serum nor DBS MFI data were enhanced when employing the OneStep assay, and the LOESS curve comparing these assays largely follows the y = x line (Additional file 3). If varying only the OneStep assay incubation times, it was found that MFI-bg signal steadily increased with increased time of incubation, and was equivalent to the standard protocol at approximately 30 to 90 min incubation time for the PfMSP1-19 and PfCSP antigens (Additional file 4).

### Differences in seroprevalence estimates using different sample types and assay protocols

As malaria serological data is typically presented in a binary fashion [an individual is +/− for antibodies against particular malaria antigen(s)], Table 2 shows the seroprevalence estimates that would be generated if applying the FMM approach to the Haiti data and defining a seropositivity signal threshold as described in Methods. A single antigen’s seroprevalence estimate was typically similar regardless of sample and protocol variations, and when comparing the average of four seroprevalence estimates (two sample types × two protocols). A notable exception to this was the estimate for PfAMA1 seropositivity for sera samples run with the OneStep protocol (10.1%) when compared to the other three estimates (27.8–34.6%).

**Table 2:** Comparison of malaria seroprevalence estimates by both assay protocols and sample types in the 2017 Haiti survey

Antigen	Serum		Dried blood spot		Average estimate % seropositive (s.d.)
	Standard (% positive)	OneStep (% positive)	Standard (% positive)	OneStep (% positive)	
PfMSP1-19	45.7	38.8	46.6	42.4	43.4 (3.5)
PvMSP1-19	5.8	8.9	7.0	7.9	7.4 (1.3)
PmMSP1-19	6.2	8.3	6.3	5.9	6.7 (1.1)
PfCSP	16.4	18.8	15.6	13.8	16.2 (2.1)
PfAMA1	27.8	10.1	27.9	34.6	25.1 (10.5)
PfLSA1	6.7	7.0	8.2	6.3	7.1 (0.82)
PfGLURP-R0	12.2	11.0	12.8	8.3	11.1 (2.4)
HRP2	11.4	13.1	8.7	13.1	11.6 (2.1)

## Discussion

Serosurveillance is increasingly becoming an attractive and economical strategy for multiple disease-specific public health programs hoping to gather information within a population regarding pathogen exposure or vaccination coverage [9, 13, 23]. As the presence of IgG antibodies typically does not distinguish past from current infection, serosurveillance provides information about exposure of a population to infectious agents over an extended period of time [24, 25]. We developed and evaluated a simplified IgG detection protocol by incubating all reagents together overnight in the assay plate, thereby greatly minimizing hands-on time required by laboratory staff: termed here the OneStep protocol. The OneStep MBA protocol augmented the detection signal for all IgG responses measured against 8 *Plasmodium* antigens when compared to a standard step-wise MBA protocol. This was found to hold true for both sera and whole blood that had been eluted from filter paper (typically referred to as dried blood spots, DBS). Serological estimates from a serosurvey in Haiti of IgG prevalence in the study population were similar regardless of protocol or sample type, indicating that the novel OneStep protocol could generate malaria exposure prevalence estimates comparable to those generated using a standard protocol employing multiple incubation steps.

When comparing the OneStep to standard immunoassays, we found the greatest increases in signal detection capacity to be in the lower- to mid-range IgG levels. Augmented assay signal may be due to the overall increased incubation time in the OneStep protocol (~ 16 h) that extends the period for specific antigen–antibody binding. Signal detection may also be boosted by the formation of IgG-reagent complexes when all immunoassay reagents are present simultaneously, and reagent association is based on binding affinity and not tandem immunoassay steps. At the highest IgG concentrations, the data suggest that beads likely reach saturation of IgG-antigen binding regardless of incubation time, and this may explain why MFI-bg signals for the PfMSP1-19, PfAMA1, and PfGLURP-R0 antigens generated by the two protocols tended to be more similar at the highest IgG concentrations. However, for the PvMSP1-19, PmMSP1-19, PfCSP, PfLSA1, and PfHRP2 antigens, the y-axis for maximum MFI signals was actually extended when using the OneStep protocol. Some stark examples of this were for the DBS data for PmMSP1-19 where max OneStep MFI-bg was 18,745 (compared to standard protocol max of 3842) and DBS data for PfHRP2 where max OneStep MFI-bg was 72,370 (compared to standard protocol max of 17,754). For IgG responses against many of the antigens, a noted limitation to the OneStep protocol was the loss of signal resolution at the higher IgG concentrations. When observing this shape of the non-parametric LOESS curves applied to scatterplots, many of the curves mirrored an exponential CDF curve which rapidly rises and eventually plateaus. If the OneStep protocol (or really any immunoassay protocol) is being used, and quantitative data is desired for analytical purposes, it is important to consider the range(s) of antibody concentration that are expected, as different protocols will provide clearer resolution to specific ranges of antibody concentrations. Additionally, no “correct” or “incorrect” total time of incubation is required by this simultaneous incubation protocol, and different laboratories can adjust according to the type of information they wish to gather.

The increase in MBA signals from the OneStep protocol were indicative of improved detection of malaria antigen-specific IgG. In applying the two-component FMM approach, the MLE estimates for the mean and variance for the first component (the putative background signal from running seronegative blood samples [22]) were generally similar between the two protocols with slight increases when using the OneStep method. This result indicates that the level of non-specific binding of blood proteins or assay reagents to the bead complex leading to background noise is largely unchanged when comparing the standard to OneStep protocols, and was further accentuated by the y = x concordance in assessing assay signals to the non-binding GST control antigen. Importantly, the mean MFI-bg estimates for the second, ‘seropositive’ FMM component were increased for all 8 malaria antigens, showing the increase in the antigen-specific IgG signal when utilizing the OneStep procedure. The OneStep procedure results in less distribution overlap between the malaria seronegative and seropositive sub-populations, potentially minimizing Type 1 (false positive) and Type 2 (false negative) misclassification errors, and the width of the indeterminate region for demarcating seropositivity [26–28]. In reality, a true study population may have two, three, or more defined signal distributions indicating different categories of past exposure history, active infection status, malnutrition, and many other potential factors [23, 29].

Regardless of how many true components exist, having a separation of other components away from the first (putative seronegative) component with reduced overlap in distributions allows MLE statistics to more efficiently provide estimates for the mean and standard deviation of estimate for the first component, and provides higher confidence in determining the true assay signal.

Malaria seroprevalence estimates for the Haiti serosurvey were similar if using different sample types (serum or DBS elution) or serology assay protocols (standard or OneStep). However, the estimate for PfAMA1 seroprevalence as generated by the OneStep protocol with serum samples (and the FMM approach) was notably lower than the three other experimental conditions. As DBS and serum samples were run in parallel, and the same bead couplings used throughout the study, the simple explanation for the dramatic difference in these estimates is that the modeled mean and variance of the first component of the OneStep sera data are markedly higher when compared to the other three FMM plots for PfAMA1. Due to these higher MLE outputs, the MFI-bg seropositivity threshold (mean + 3SD) was calculated to be much higher, and thus, some samples even with a high assay signal were not considered to have a MFI-bg signal “positive” for IgG against PfAMA1. This shows the susceptibility of forcing the mixture model to define two components, and comparison of seroestimates and cutoff determination schemes with various sample types and protocols should be investigated further. Overall, consistency among malaria seroprevalence estimates under different MBA conditions would indicate these estimates are accurate approximations of the true point prevalence.

## Conclusion

When compared to a standard MBA protocol, the OneStep serology protocol with samples and reagents incubated simultaneously overnight generated comparable estimates of overall seroprevalence for most antigens tested in a Haitian malaria survey, and may offer an efficient and flexible approach to large-scale serosurveillance studies. As malaria serosurveys become larger and more widely employed, options for the timely collection of laboratory data will be needed to allow a fast transition from sample collection to analyses.

## Supplementary information

**Additional file 1.** Fittings to Two-Component Finite Mixture Models for Antigen Data Collected by both Assay Protocols and Sample Types for Low Seroprevalence Antigens. Histograms are displayed for log-transformed MFI-bg values for the four least immunogenic malaria antigens as fit to a two-component finite mixture model. On each panel, estimates for lognormal mean and variance are displayed for the two components.

**Additional file 2.** Maximum Likelihood Estimates from Finite Mixture Model Comparing Standard to OneStep Protocols and Serum to Blood Elutions from Haiti Samples.

**Additional file 3.** Scatterplots for assay signal for generic GST antigen for sera and dried blood spot samples when comparing standard and OneStep assay protocols for malaria antigens.

**Additional file 4.** Increasing Incubation Times for the OneStep Assay Increases MFI-bg Assay Signal for Selected Malaria Antigens. Hyperimmune serum for malaria antigens was serially-diluted and incubated for 15, 30, 60, or 90 min with OneStep protocol, or assayed with standard protocol.

## Data Availability

All data is available upon reasonable request.

## Abbreviations

AMA1: apical membrane antigen 1
CSP: circumsporozoite protein
DBS: dried blood spot
ELISA: enzyme-linked immunosorbent assay
HRP2: histidine-rich protein 2
FMM: finite mixture model
GST: glutathione-*S*-transferase
GLURP-R0: glutamate-rich protein R0 fragment
LOESS: locally weighted regression
LSA1: liver stage antigen 1
MBA: multiplex bead assay
MFI: median fluorescence intensity
MLE: maximum likelihood estimate
MSP1: merozoite surface protein 1
SD: standard deviation
