# Supplementary figures and images for: High-throughput malaria serosurveillance using a one-step multiplex bead assay
Source: Malar J. 2019 Dec 4;18:402. doi: 10.1186/s12936-019-3027-0 (PMC6894145; doi:10.1186/s12936-019-3027-0)

Additional File 1

Percent

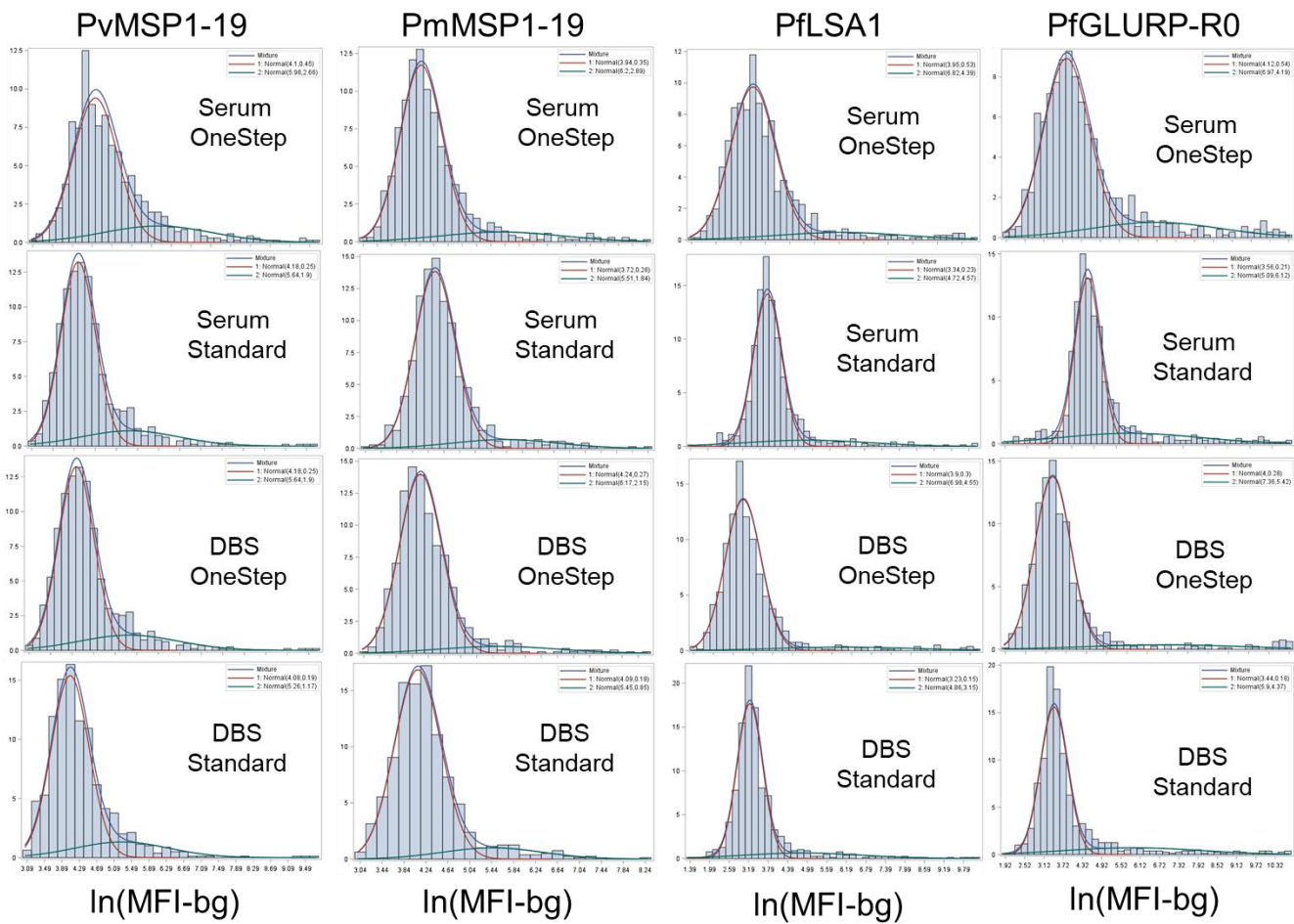

Supplement: Supplementary file 1 — Additional file 1. Fittings to Two-Component Finite Mixture Models for Antigen Data Collected by both Assay Protocols and Sample Types for Low Seroprevalence Antigens. Histograms are displayed for log-transformed MFI-bg values for the four least immunogenic malaria antigens as fit to a two-component finite mixture model. On each panel, estimates for lognormal mean and variance are displayed for the two components. [file 12936_2019_3027_MOESM1_ESM.pdf]

Additional File 3

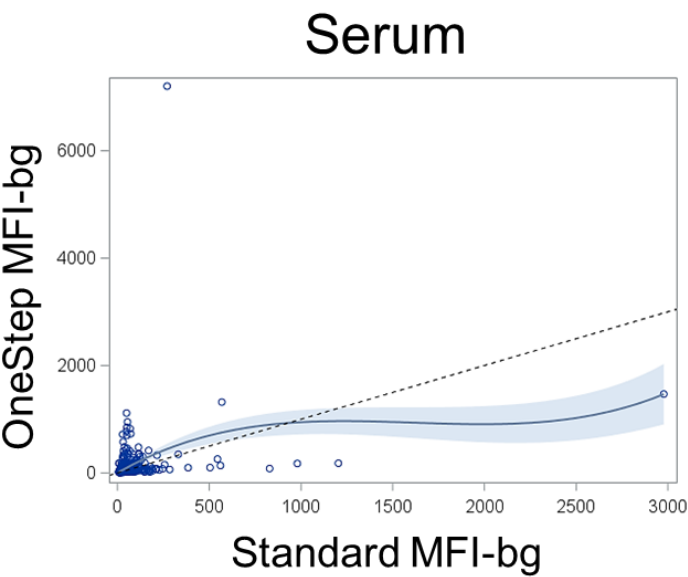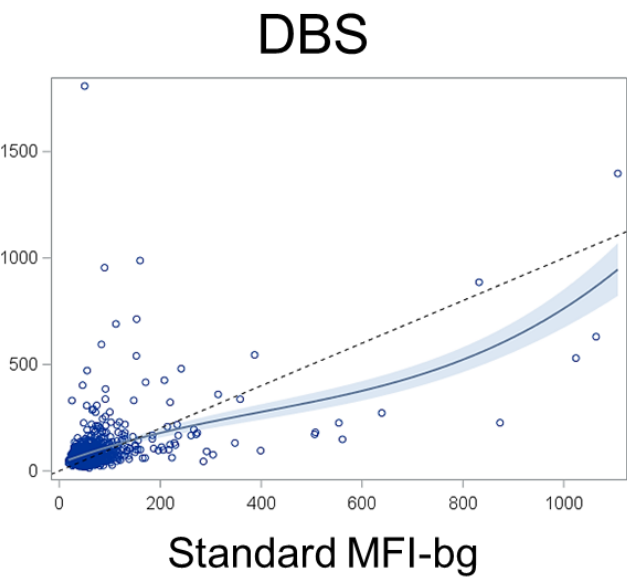

Supplement: Supplementary file 3 — Additional file 3. Scatterplots for assay signal for generic GST antigen for sera and dried blood spot samples when comparing standard and OneStep assay protocols for malaria antigens. [file 12936_2019_3027_MOESM3_ESM.pdf]

Additional File 4

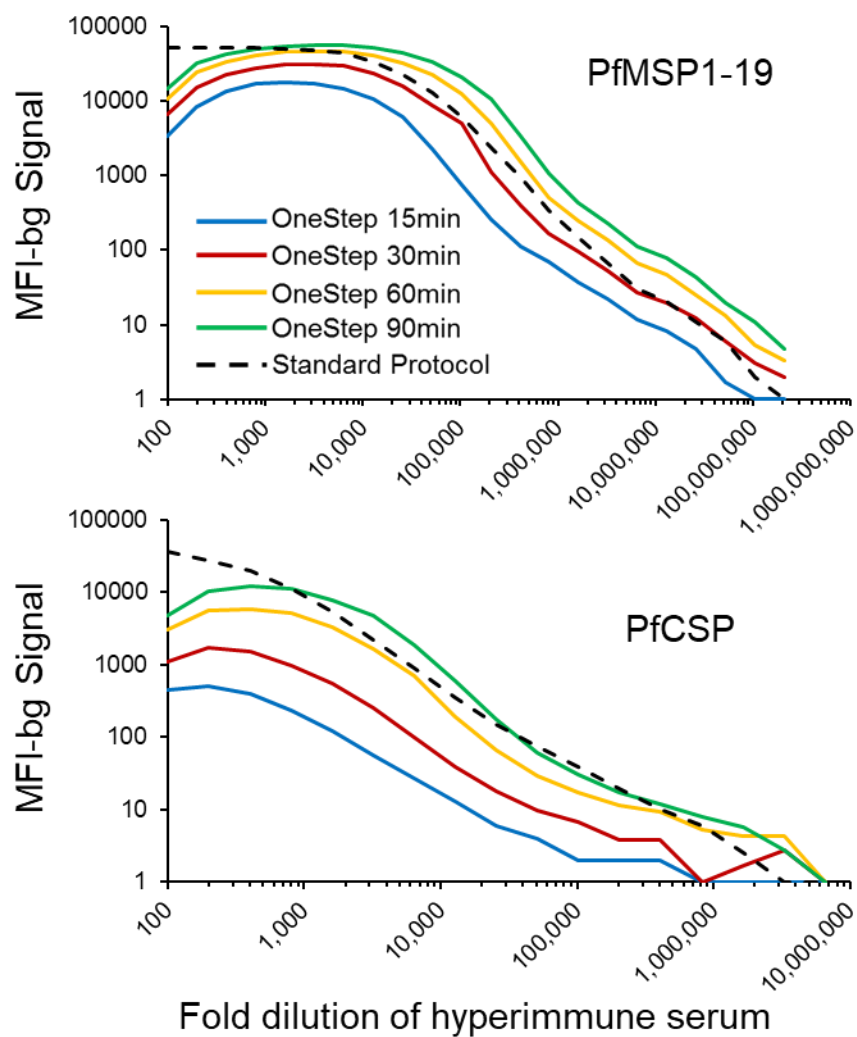

Supplement: Supplementary file 4 — Additional file 4. Increasing Incubation Times for the OneStep Assay Increases MFI-bg Assay Signal for Selected Malaria Antigens. Hyperimmune serum for malaria antigens was serially-diluted and incubated for 15, 30, 60, or 90 min with OneStep protocol, or assayed with standard protocol. [file 12936_2019_3027_MOESM4_ESM.pdf]
