# Supplementary material for: High-throughput malaria serosurveillance using a one-step multiplex bead assay
Source: Malar J. 2019 Dec 4;18:402. doi: 10.1186/s12936-019-3027-0 (PMC6894145; doi:10.1186/s12936-019-3027-0)
Supplement: Supplementary file 2 — Additional file 2. Maximum Likelihood Estimates from Finite Mixture Model Comparing Standard to OneStep Protocols and Serum to Blood Elutions from Haiti Samples. [file 12936_2019_3027_MOESM2_ESM.pdf]

Additional File 2

Antigen	Sample Type	Protocol	Component 1 lognormal mean (variance)	Component 2 lognormal mean (variance)	Δ lognormal means C1 vs C2
**PfMSP1-19**	Serum	Standard	3.6 (0.50)	8.1 (3.64)	4.5
		OneStep	4.7 (2.65)	10.5 (0.29)	5.8
	Blood Elution	Standard	3.5 (0.25)	7.3 (4.21)	3.8
		OneStep	4.0 (0.71)	10.1 (1.43)	6.1
**PvMSP1-19**	Serum	Standard	4.2 (0.25)	5.6 (1.9)	1.4
		OneStep	4.1 (0.45)	6.0 (2.66)	1.9
	Blood Elution	Standard	4.1 (0.19)	5.3 (1.17)	1.2
		OneStep	4.2 (0.25)	5.6 (1.9)	1.4
**PmMSP1-19**	Serum	Standard	3.7 (0.26)	5.5 (1.84)	1.8
		OneStep	3.9 (0.35)	6.2 (2.89)	2.3
	Blood Elution	Standard	4.1 (0.18)	5.5 (0.85)	1.4
		OneStep	4.2 (0.27)	6.2 (2.15)	2.0
**PfCSP**	Serum	Standard	3.7 (0.45)	6.1 (3.28)	2.4
		OneStep	4.1 (0.66)	7.5 (4.14)	3.4
	Blood Elution	Standard	3.5 (0.31)	5.7 (2.85)	2.2
		OneStep	4.1 (0.38)	7.4 (4.41)	3.3
**PfAMA1**	Serum	Standard	4.1 (0.84)	7.8 (2.85)	3.7
		OneStep	5.4 (3.04)	10.3 (0.28)	4.9
	Blood Elution	Standard	3.9 (0.49)	7.1 (3.48)	3.2
		OneStep	4.5 (0.85)	9.6 (2.05)	5.1
**PfLSA1**	Serum	Standard	3.4 (0.23)	4.7 (4.57)	1.3
		OneStep	4.0 (0.53)	6.8 (4.39)	2.8
	Blood Elution	Standard	3.2 (0.15)	4.9 (3.15)	1.7
		OneStep	3.9 (0.30)	7.0 (4.55)	3.1
**PfGLURP-R0**	Serum	Standard	3.6 (0.21)	5.1 (6.12)	1.5
		OneStep	4.1 (0.54)	7.0 (4.19)	2.9
	Blood Elution	Standard	3.4 (0.18)	5.9 (4.37)	2.5
		OneStep	4.0 (0.28)	7.4 (5.42)	3.4
**HRP2**	Serum	Standard	3.9 (0.34)	6.0 (2.98)	2.1
		OneStep	4.4 (0.59)	7.1 (4.02)	2.7
	Blood Elution	Standard	4.0 (0.24)	5.7 (1.99)	1.7
		OneStep	4.3 (0.3)	7.1 (3.62)	2.8
